# Outcomes and Treatment Options for Duodenal Adenocarcinoma: A Systematic Review and Meta-Analysis

**DOI:** 10.1245/s10434-018-6567-6

**Published:** 2018-06-26

**Authors:** Laura L. Meijer, Anna J. Alberga, Jacob K. de Bakker, Hans J. van der Vliet, Tessa Y. S. Le Large, Nicole C. T. van Grieken, Ralph de Vries, Freek Daams, Barbara M. Zonderhuis, Geert Kazemier

**Affiliations:** 1 Department of Surgery, Cancer Center Amsterdam, VU University Medical Center, Amsterdam, The Netherlands; 2 Department of Medical Oncology, Cancer Center Amsterdam, VU University Medical Center, Amsterdam, The Netherlands; 3 Laboratory for Experimental Oncology and Radiobiology, Academic Medical Center, Amsterdam, The Netherlands; 4 Department of Pathology, Cancer Center Amsterdam, VU University Medical Center, Amsterdam, The Netherlands; 5 Medical Library, VU University Amsterdam, Amsterdam, The Netherlands

## Abstract

**Background:**

Duodenal adenocarcinoma (DA) is a rare tumor for which survival data per treatment modality and disease stage are unclear. This systematic review and meta-analysis aims to summarize the current literature on patient outcome after surgical, (neo)adjuvant, and palliative treatment in patients with DA.

**Methods:**

A systematic search was performed according to the preferred reporting items for systematic reviews and meta-analyses guidelines, to 25 April 2017. Primary outcome was overall survival (OS), specified for treatment strategy or disease stage. Random-effects models were used for the calculation of pooled odds ratios per treatment modality. Included papers were also screened for prognostic factors.

**Results:**

A total of 26 observational studies, comprising 6438 patients with DA, were included. Of these, resection with curative intent was performed in 71% (range 53–100%) of patients, and 29% received palliative treatment (range 0–61%). The pooled 5-year OS rate was 46% after curative resection, compared with 1% in palliative-treated patients (OR 0.04, 95% confidence interval [CI] 0.02–0.09, *p* < 0.0001). Both segmental resection and pancreaticoduodenectomy allowed adequate assessment of lymph node involvement and resulted in similar OS. Lymph node involvement correlated with worse OS (pooled 5-year survival rate 21% for nodal metastases vs. 65% for node-negative disease; OR 0.17, 95% CI 0.11–0.27, *p* < 0.0001). In the current literature, no survival benefit for adjuvant therapy after curative resection was found.

**Conclusion:**

Resection with curative intent, either pancreaticoduodenectomy or segmental resection, and lack of nodal metastases, favors survival for DA. Further studies exploring multimodality (neo)adjuvant therapy are warranted to investigate their benefit.

**Electronic supplementary material:**

The online version of this article (10.1245/s10434-018-6567-6) contains supplementary material, which is available to authorized users.

Duodenal adenocarcinoma (DA) is a rare tumor representing approximately 0.5% of all gastrointestinal tumors, although it accounts for more than 50% of small bowel adenocarcinomas (SBAs), and in many studies outcomes for all SBAs are grouped together.^1^–^3^ Due to the location of the duodenum in the gastrointestinal tract, molecular similarities, and possibly similar phenotypic carcinogenesis, DA is often compared with colorectal cancer (CRC).^4^^,^^5^ However, tumor location is a known prognostic factor both in CRC and SBAs.^6^^,^^7^ Therefore, there is a clear need to report outcomes per treatment modality of DA as a unique entity.

Resection of the primary tumor is the only curative treatment option for DA.^8^^,^^9^ Pancreaticoduodenectomy is often performed to ensure radical resection and adequate lymphadenectomy. For distal DA, segmental duodenal resection is reported to be a justified alternative, but this is often disputed because of the presumed inadequate lymph node clearance compared with pancreaticoduodenectomy.^10^–^14^ In a palliative setting, a gastrojejunal bypass procedure is commonly performed, although the influence on overall survival (OS) is unclear.^13^

Chemo(radiation) therapy has been proposed to further enhance long-term survival after curative resection and as palliative therapy for advanced tumor stages. Given the similarities between SBA and CRC, interchangeable radio- and chemotherapeutic strategies are frequently offered to patients with DA.^5^^,^^15^^,^^16^ In CRC, palliative chemotherapy for metastatic disease and adjuvant chemotherapy for resected node-positive CRC have become standard of care.^17^–^19^ Therefore, the effect of similar regimens has been investigated for adjuvant and palliative treatment in DA, including fluorouracil-based chemotherapy, often combined with a platinum analog or radiation therapy.^20^–^22^ In addition, neoadjuvant therapy is implemented for gastrointestinal cancers, such as esophageal carcinoma, but not yet investigated for DA.^23^ Considering the shift towards more (neo)adjuvant treatment strategies, this might also be beneficial for DA.

Practical guidelines for the treatment of DA are lacking and controversy exists about the most effective chemo(radiation) therapy regimens, in both the (neo)adjuvant and palliative settings.^8^^,^^24^ The aim of this review of the literature and meta-analysis is to describe the outcomes of DA after curative and palliative treatment strategies, including optimal type of resection and the value of (neo)adjuvant therapy, and to determine the role of prognostic factors.

## Methods

This systematic review was conducted and reported in accordance with the preferred reporting items for systematic reviews and meta-analysis (PRISMA) guidelines.^25^ A systematic literature search was performed in the PubMed, EMBASE, and Wiley/Cochrane Library electronic databases from inception to 25 April 2017. The following terms were used (including synonyms and closely related words) as index terms or free-text words: ‘adenocarcinoma’, ‘duodenum’, ‘ampulla of Vater’, and ‘therapeutics’. All studies reporting on survival for histologically confirmed DA or intestinal-type ampullary adenocarcinoma (IAA) were eligible for inclusion. The reported survival rates had to be specified either per intervention group or per disease stage.^26^ Statistical analyses included pooling of the studies to compare the number of events after 5 years by the odds ratios (ORs) for death with their associated 95% confidence interval (CI). The Mantel–Haenszel method was used to calculate the weighted pooled OR and their associated 95% CIs for dichotomous data under the random effects model.^27^^,^^28^ Statistical heterogeneity was estimated using the Cochrane’s Q and *I*^2^ statistics,^29^ and the Newcastle–Ottawa quality assessment was implemented to assess the quality and risk of bias of the included studies.^30^ The full search strategies for all databases, eligibility criteria, inclusion and exclusion criteria, data collection process and analysis, and assessment of methodological quality can be found in the electronic supplementary material. Two investigators (LM and AA) independently reviewed the literature and conducted study selection. Data extraction and analysis were conducted by three investigators (LM, AA and JB). Discordant judgment was resolved by discussion and consensus.

## Results

### General Characteristics of the Included Studies

**Fig. 1:**
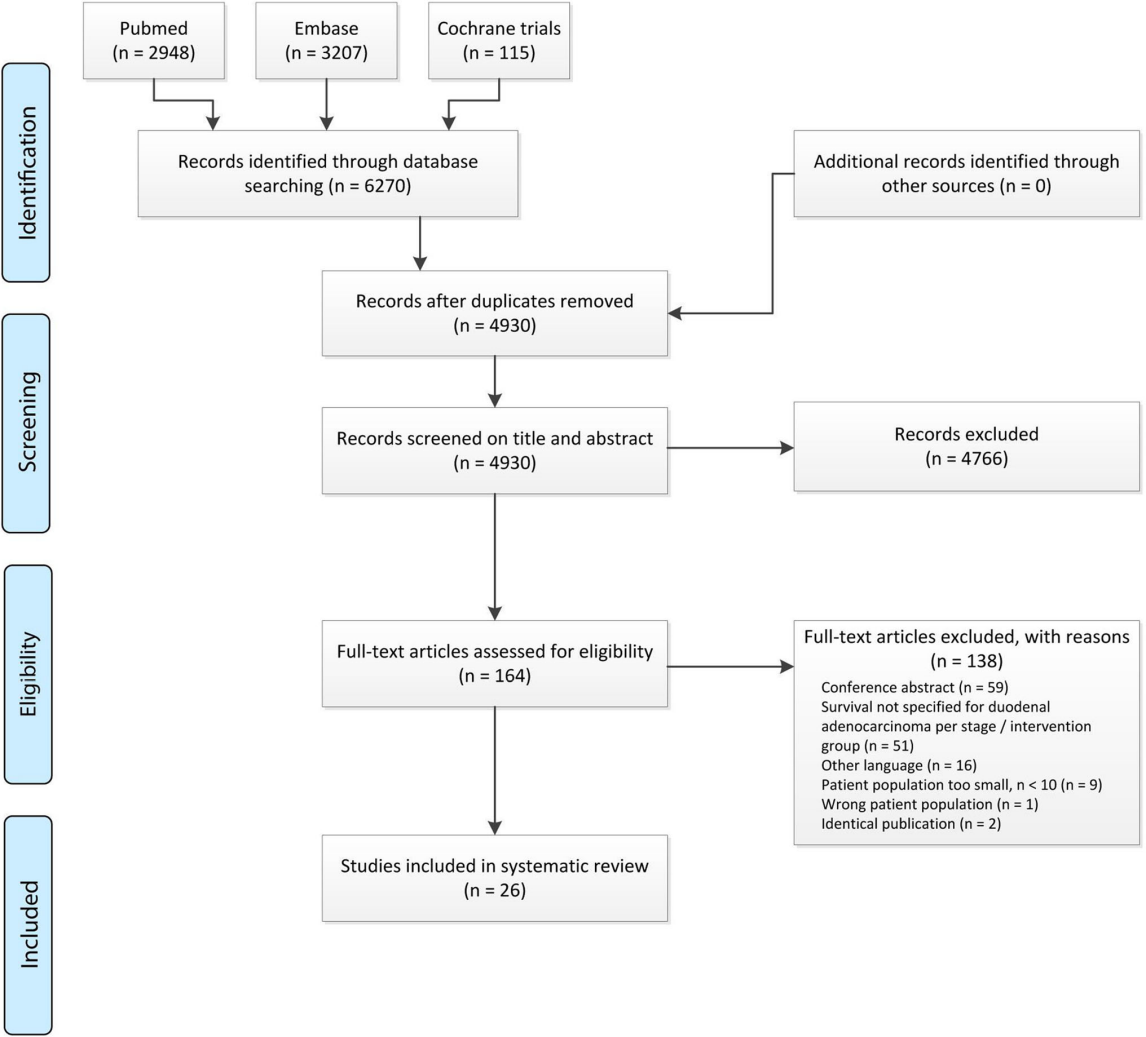
Article selection process

After reviewing articles for title, abstract and full text, 26 studies met the inclusion criteria and were included for critical appraisal (Fig. 1). No studies describing patients with IAA met the inclusion criteria, thus only those describing patients with DA were eligible for further analyses.

As shown in Table 1, 23 retrospective cohort studies, 2 prospective cohort studies, and 1 case–control study comprising 6438 patients were included. The weighted mean age was 63 years and 53% of patients were male. DA was classified according to American Joint Committee on Cancer (AJCC) 5th, 6th, or 7th edition; six studies did not report the edition of AJCC classification. Given the minor differences between the AJCC staging editions, subdivision within disease stage was not further taken into account in our analyses.

**Table 1:** General characteristics of the included studies

Author (year of publication)	*N*	Study period	Trial setting	Country	Age (SD)^a^	Males [*n* (%)]	Follow-up (months, median)	Interventions for which survival is reported	Type of survival outcome studied (in years)	Tumor location				AJCC edition
										D1 + D1/2	D2 + D2/3	D3 + D3/4	D4 + Treitz	
Bakaeen^31^	101	1976–1996	RCS	USA	63 (14)	51 (50)	39.6^b^	CR, PT	OS-3,5	3	50	9	6	5
Bhatti^47^	12	1999–2012	RCS	Pakistan	55 (10)	8 (67)	–	CR	M, OS-5	–	–	–	–	7
Buchbjerg^2^	71	1997–2012	RCS	Denmark	67 (13)	43 (61)	–	CR, PT	M, OS-1,3,5	7	36	19	10	5
Cecchini^32^	169	1982–2010	RCS	USA	62 (13)	93 (55)	26.5	CR, PT	M, OS-3,5	10	72	10	11	7
Cloyd^43^	1611	1988–2010	RCS	USA	–	745 (46)	41.9	CR	M, OS-5	–	–	–	–	6
Ecker^50^	3122	1998–2012	CCS	USA	66 (14)	1683 (54)	79.2	Adj. CRTx, adj. CTx	M, OS-5	–	–	–	–	–
Han^10^	32	1990–2006	RCS	China	56 (7)	19 (59)	106	CR, PT	OS-1,3,5	2	8	17	5	6
Hung^33^	23	1994–2005	RCS	Taiwan	68 (12)	15 (65)	15.1	CR, PT	M, OS-1,3,5	9	14	–	–	–
Hurtuk^44^	52	1984–2005	RCS	USA	65 (12)	36 (69)	24	CR, PT	M, OS-3,5	–	–	–	–	–
Jiang^34^	201	1999–2015	RCS	China	55 (10)	78 (61)^b^	20	CR, PT	M, OS-1,3,5	5	113	9	4	7
Kaklamanos^12^	63	1978–1988	RCS	USA	61 (18)	33 (52)	–	CR, PT	M, OS-5	7	41	–	4	5
Kawahira^35^	21	1977–2007	RCS	Japan	61 (–)	11 (52)	–	CR, PT	M, OS-1,3,5	–	–	–	–	7
Kelsey^22^	32	1975–2005	RCS	USA	57 (11)	23 (72)	32	CR, CR + adj. CRTx	OS-5	0	14	11	7	–
Kim^45^	24	1991–2002	RCS	South Korea	58 (11)	14 (58)	32	CR, CR + adj. CRTx	OS-5	–	–	–	–	6
Kim^36^	50	1995–2010	RCS	South Korea	61 (11)	35 (70)	–	CR, PT	M, OS-3,5	9	24	2	1	7
Lee^37^	53	1995–2007	RCS	South Korea	60 (10)	33 (62)	41.7	CR, PT	OS-3,5	6	30	13	4	–
Lee^38^	76	1999–2009	RCS	South Korea	56 (11)	55 (72)	–	CR, PT	M, OS-1,3,5	–	41	7	–	7
Liang^41^	36	1993–2010	RCS	Taiwan	64 (13)	24 (67)	41	CR	M, OS-3,5	8	25	2	1	7
Malleo^40^	37	2000–2009	RCS	Italy	57 (11)	21 (57)	25	CR, PT	M, OS-5	–	25	12	–	7
Onkendi^11^	124	1994–2009	RCS	USA	65 (14)	75 (59)	–	CR, PT	M, OS-2,5,10	8	73	24	15	7
Poultsides^42^	122	1984–2006	RCS	USA	67 (14)	66 (54)	33	CR, CR + adj. CRTx	OS-5,10	–	–	–	–	7
Sarela^46^	137	1983–2001	PCS	USA	63 (11)	75 (55)	36	CR	OS-5,10	–	–	–	–	5
Solaini^24^	178	2000–2013	PCS	UK	61 (4)	101 (57)	39	CR, PT	M, OS-1,3,5	25	94	29	12	7
Struck^48^	30	1989–2006	RCS	USA	61 (10)	22 (73)	15.2	CR	M, OS-1,5	–	–	–	–	6
Swartz^49^	14	1994–2003	RCS	USA	53 (9)	10 (71)	42	CR, CR + adj. CTx	M, OS-5	–	–	–	–	6
Tocchi^39^	47	1980–2000	RCS	Italy	58 (8)	26 (45)	24	CR, PT	M, OS-5	–	–	37	10	–
All studies	6438				63^c^	3395 (53)				99	660	201	90	

*n* number of patients included, *PCS* prospective cohort study, *RCS* retrospective cohort study, *CCS* case–control study, *SD* standard deviation, *AJCC* American Joint Committee on Cancer, *M* median survival reported, *OS* overall survival, *CR* resection with curative intent (R0/R1 resection, pancreaticoduodenectomy or segmental resection), *PT* palliative treatment (R2 resection, bypass, stent placement, palliative or supportive treatment), – Indicates not reported

^a^Age: mean in years (range in years)

^b^Only reported for resection with curative intent

^c^Weighted mean

### Survival After Resection with Curative Intent and Palliative Treatment

**Fig. 2:**
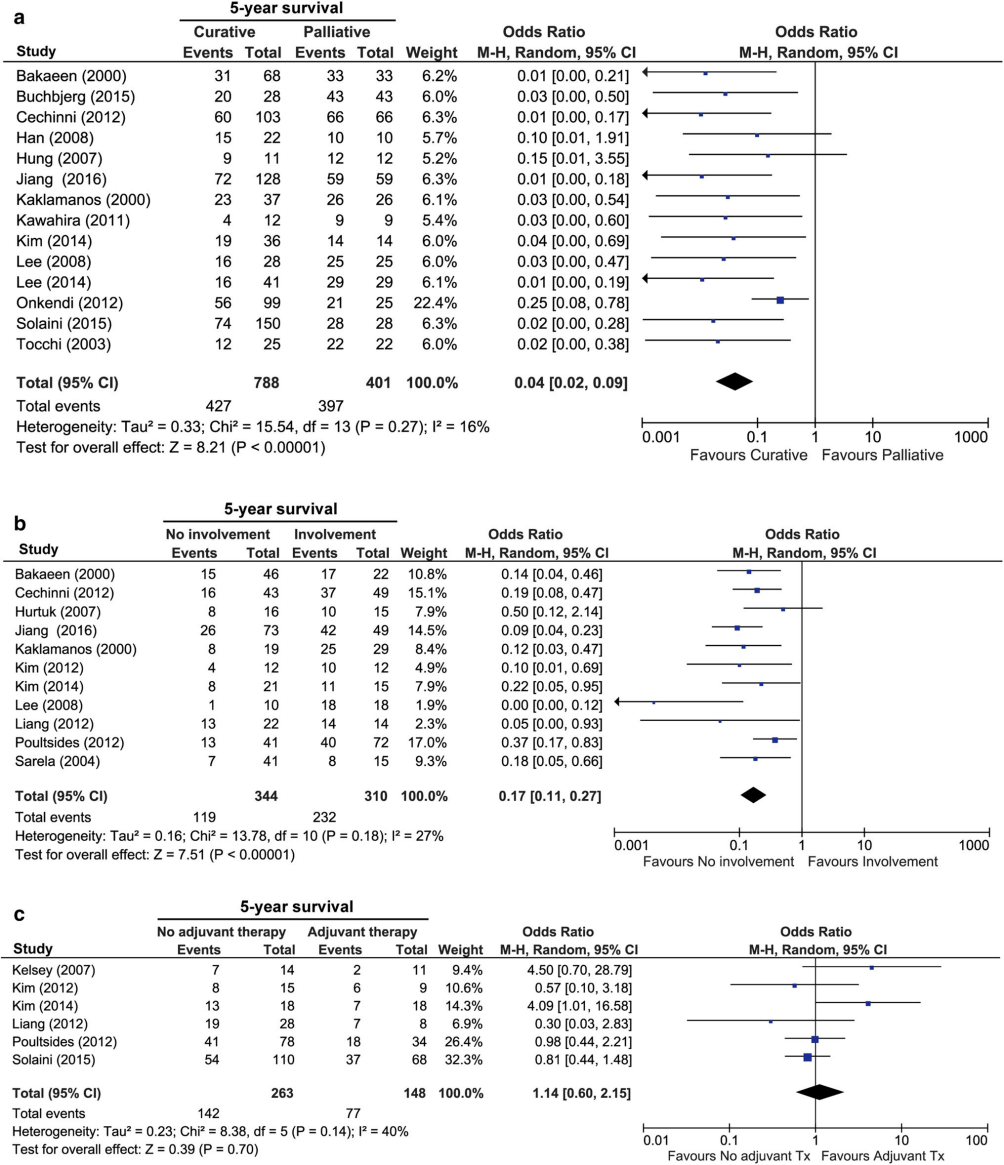
Forest plots of the meta-analyses of studies examining the effect of **a** resection with curative intent to palliative treatment; **b** nodal involvement; and **c** adjuvant therapy on overall survival. The odds ratios associated with the 5-year survival and pooled overall odds ratio for each of the studies are shown. The M-H random-effects model was used for meta-analysis. Values in brackets are 95% CIs. *M*–*H* Mantel–Haenszel, *CI* confidence interval, *df* degrees of freedom, *Tx* treatment

A median of 71% (range 53–100%) of patients underwent resection with curative intent and 29% (range 0–61%) received palliative treatment (“Appendix” section). Overall, the pooled 5-year OS of DA was 36%, with a median OS of 31 months. In the 14 studies comparing curative and palliative treatment, the pooled 5-year survival rate was significantly longer when treatment with curative intent was feasible (46 vs. 1%, respectively; OR 0.04, 95% CI 0.02–0.09; *I*^*2*^ = 16%, *p* < 0.0001) (Fig. 2a).^2^^,^^10^–^12^^,^^24^^,^^31^–^39^ Eight studies reported no influence of age and sex on OS in the total study population.^24^^,^^36^–^42^ Pooling of studies to estimate survival per disease stage could not be performed due to the lack of specification of survival per disease stage. Only three studies specified survival rates.^36^–^38^^,^^41^

### Curative Resection

A total of eight studies, comparing survival of patients treated with either pancreaticoduodenectomy or segmental resection, were eligible for comparison.^10^–^12^^,^^22^^,^^32^^,^^34^^,^^39^^,^^43^ All these studies reported no significant differences in survival comparing segmental resection with pancreaticoduodenectomy. Two studies reported a greater number of lymph nodes sampled and more positive lymph nodes removed in patients undergoing pancreaticoduodenectomy, compared with segmental resection.^11^^,^^43^ However, this was not confirmed by another study and did not affect OS.^12^

Six patients with either hepatic or (retro)peritoneal metastases underwent resection of metastases concomitantly with resection of the primary duodenal tumor.^2^^,^^22^^,^^32^ Cecchini et al. reported a 3-year survival of 68% and a 5-year survival of 57% after metastases resection. No comparisons between metastasectomy and systemic therapy were made.^32^

### The Influence of Nodal Metastases on Survival Rates

The most commonly reported prognostic factor was the involvement of lymph nodes after resection. Eleven studies compared the 5-year survival rate for patients with lymph node involvement (N +) with those without lymph node involvement (N0). The pooled 5-year survival rate was 65% for N0, compared with 21% for N + , resulting in significantly shorter survival when involvement of lymph nodes was present (OR 0.17, 95% CI 0.11–0.27, *p* < 0.0001) (Fig. 2b).^12^^,^^31^^,^^32^^,^^34^^,^^36^^,^^37^^,^^41^^,^^42^^,^^44^–^46^ Lymph node involvement remained an independent prognostic factor in most studies after correction for other clinicopathological factors, including tumor size, differentiation grade, and disease stage.^12^^,^^24^^,^^31^^,^^34^^,^^37^^,^^41^^,^^42^^,^^45^^,^^46^

### Adjuvant and Neoadjuvant Therapy

Six studies investigated the 5-year OS for any type of adjuvant therapy compared with no adjuvant therapy.^22^^,^^24^^,^^36^^,^^41^^,^^42^^,^^45^ There was no difference in the pooled 5-year OS for any type of adjuvant therapy and control groups (48 vs. 46%, respectively; OR 1.14, 95% CI 0.60–2.15, *I*^2^ = 40%) (Fig. 2c). No specific analysis stratified per treatment modality could be made due to heterogeneous groups in the included studies and insufficient information regarding the selection of patients for adjuvant therapy. Only two studies corrected for nodal metastases and found no survival benefit for adjuvant therapy after correction for lymph node involvement.^42^^,^^45^

Many studies described various agents for adjuvant treatment, either without reporting the effect on survival^10^^,^^38^^,^^40^^,^^47^ or without reporting any significant effect.^11^^,^^31^^,^^32^^,^^34^^,^^38^^,^^43^^,^^48^^,^^49^ Most studies did not specifically report the influence of adjuvant therapy for patients diagnosed with nodal involvement.^11^^,^^12^^,^^22^^,^^24^^,^^31^^,^^32^^,^^34^^,^^36^–^38^^,^^40^^,^^41^^,^^43^^,^^48^^,^^50^

Ten studies, compromising 203 patients, specified the adjuvant chemotherapeutic regimens.^10^^,^^22^^,^^31^^,^^32^^,^^36^^,^^38^^,^^42^^,^^45^^,^^48^^,^^49^ Of these, a total of 199 patients (98%) were treated with fluorouracil-based chemotherapeutic regimens (e.g. 5-fluorouracil or capecitabine), either as monotherapy or combined with a second agent, such as a platinum-based compound.^36^^,^^38^ Adjuvant radiation therapy in combination with chemotherapy was administered to 74% of all patients treated with adjuvant therapy.^22^^,^^31^^,^^32^^,^^36^^,^^42^^,^^45^^,^^48^^,^^49^ Only one study compared the use of adjuvant chemoradiotherapy with adjuvant chemotherapy in 1028 patients, and no significant survival advantage was observed between both groups after matched analysis.^50^

In this review, five studies comprising a total of 117 patients mentioned the use of preoperative chemotherapy and/or radiotherapy.^2^^,^^22^^,^^32^^,^^38^^,^^50^ These studies either did not investigate benefit or reported no improvement in OS. Interestingly, one study reported a pathologic complete response in 18% of patients after preoperative chemoradiation.^22^

### Palliative Treatment

Palliative treatment was described in 17 studies comprising 495 patients.^2^^,^^10^–^12^^,^^24^^,^^31^–^40^^,^^44^^,^^46^ Of those patients, 309 were treated with bypass surgery (i.e. gastrojejunostomy in 32%, and combined with biliary bypass in 8%). The influence of palliative bypass surgery on OS was not reported. Data of non-surgical palliative treatment were scarce. Four studies described a total of 46 patients receiving palliative chemotherapy, but regimens were not further specified.^2^^,^^12^^,^^32^^,^^33^ Palliative radiotherapy or chemoradiation therapy was described in three patients.^22^^,^^33^ The influence of systemic treatment on survival was not reported.

### Quality of the Included Studies

**Fig. 3:**
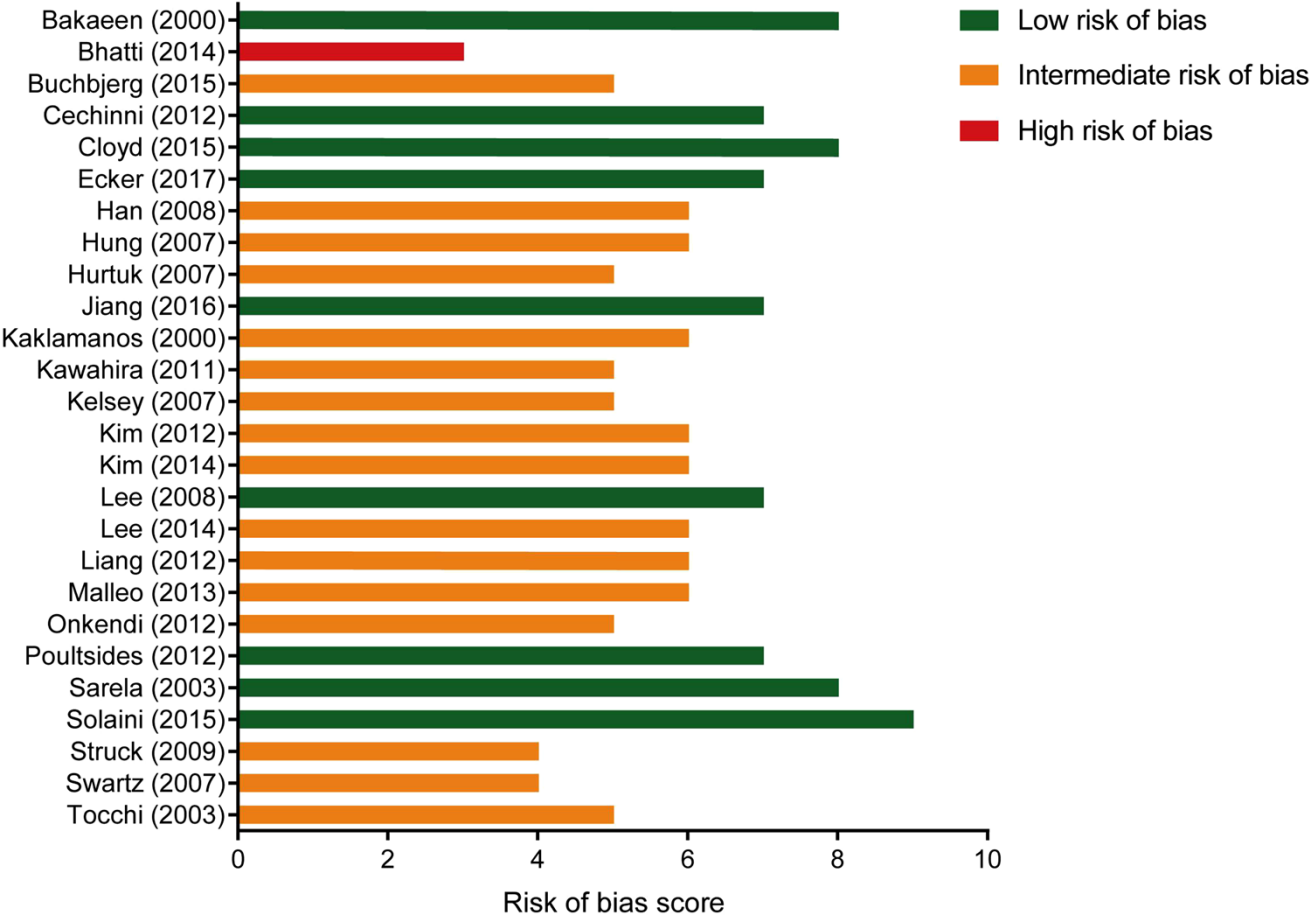
Quality assessment of the included studies based on the Newcastle–Ottawa quality assessment scale for case–control studies. The maximum score is 9

An evaluation of the quality of the included studies is presented in Fig. 3. Nine studies scored a low risk of bias, while 16 studies scored an intermediate risk of bias, and one scored a high risk of bias. As evidenced by the relevant findings in this meta-analysis, the quality of the included studies was mainly compromised by clinical incomparability of both factors that could influence survival, such as age, sex, and tumor stage, as well as limited therapy specifications. In addition, adjusted estimates of OS were insufficiently reported to be included for our meta-analysis.

## Discussion

This systematic review of the literature and meta-analysis shows a clear survival benefit for patients with DA after curative surgical resection, compared with palliative-treated patients. Both segmental duodenal resection and pancreaticoduodenectomy allow for adequate removal of lymph nodes and result in similar OS when negative resection margins can be achieved. Involvement of lymph nodes is a negative prognostic factor for survival and results in a significantly lower 5-year OS rate than node-negative disease. The included studies show no associated survival benefit for the use of any type of adjuvant therapy for DA, although this remains debatable due to the inequality of regimes used and insufficient patient stratification. No consensus regarding palliative treatment was found. Rarity of the tumor precluded a reliable assessment of the outcome of DA for each disease stage as the number of patients included per study was often low and staging was poorly specified.

To adequately attribute the relevance of lymph node metastases to prognosis, the number of involved lymph nodes and the pattern of regional lymph node spread is important.^14^^,^^26^^,^^46^ Lymphatic spread pattern is different among locations of DA, with lymphatic drainage of the distal portions of the duodenum ending mostly in the small bowel mesentery. These lymphatic basins are not removed with lymphadenectomy performed during pancreaticoduodenectomy, suggesting no additive effect of extended lymph node resection on survival for distal tumors.^31^ Although some still advocate pancreaticoduodenectomy for all DA tumors,^14^ our results emphasize that segmental resection is the resection of choice for distal DA whenever radical resection margins can be accomplished.^10^–^12^^,^^22^^,^^32^^,^^34^^,^^39^^,^^43^

Both DA and IAA display an intestinal histopathological phenotype and reported survival rates appear to be comparable.^51^ Notably, periampullary tumors with an intestinal histopathological phenotype with no lymph node metastasis showed an exceptionally good prognosis compared with pancreaticobiliary tumors.^52^ This provides rationale for similar treatment strategies for DA and IAA since therapies may be more adequately addressed based on histopathological classifications.^53^ Remarkably, no studies reporting treatment of IAA met the predefined inclusion criteria due to low numbers and thus these could not be incorporated for critical appraisal.^54^ Besides a histopathological approach, a targeted approach might be even more suitable to optimize treatment allocation for patients with DA and IAA. Recent comprehensive studies gained insight into the biology of periampullary cancers. Using genomic analysis, including driver gene mutations, they underlined the resemblance of IAA to intestinal tumors, such as CRC, while distinct alterations were also found.^55^^,^^56^ Periampullary tumors and DA showed overlapping alterations in pathway genes, irrespective of tumor origin.^56^ These new insights support the clinical treatment of these tumor types as a unique entity and endeavor personalized treatment guided by genomic analysis.

Since local treatment of colorectal liver metastases prolongs survival, it has been suggested that patients with solitary or oligocentric hepatic metastases from DA and IAA should be considered for resection of metastases.^32^^,^^57^ In contrast to resection of liver metastases of pancreaticobiliary lesions, intestinal-type tumors show an impressively better survival.^57^ Although too little experience exists to compare survival after resection of metastases to local or systemic treatment, first results regarding hepatic or (retro)peritoneal metastatic resection in DA show an encouraging improvement in OS.^32^

In this study, adjuvant chemo(radiation) therapy did not result in a proven survival benefit,^22^^,^^24^^,^^31^^,^^34^^,^^36^^,^^41^^,^^48^^,^^50^ even after correction for nodal metastases.^42^^,^^45^ In the latter two studies, adjuvant therapy resulted in similar survival rates compared with no adjuvant therapy, despite a higher prevalence of lymph node involvement in the adjuvant therapy group.^45^ Although further subgroup assessment is needed, these results could indicate a selection bias of patients for adjuvant therapy and might suggest a benefit for administration of adjuvant therapy in patients with worse prognosis. Adequate data on disease stage are essential to make definite conclusions as to whether adjuvant chemotherapy can be beneficial and to tailor treatment strategies accordingly. In comparison, adjuvant chemotherapy prolongs survival in node-positive disease (stage III), but not in node-negative disease (stage II) CRC.^58^ Since this stratification has not yet been thoroughly investigated for DA, the true value of adjuvant therapy based on the current literature remains unknown. The ongoing open-label randomized controlled BALLAD trial (NCT02502370) aims to answer this challenging clinical question by evaluating the potential benefit of two different adjuvant chemotherapy schedules for patients with resected stage I, II, and III SBA.

Growing evidence indicates favorable outcomes for combined modality therapies in CRC.^59^^,^^60^ Subgroup analyses in CRC emphasize the importance of location and molecular subtypes to further stratify patients for optimal therapy outcomes.^61^^,^^62^ These experiences gained in the CRC field in the past 20 years may also be explored in DA.^17^^,^^56^ However, in DA, monotherapy with fluorouracil-based regimens is still commonly offered in the clinic, with no clear evidence of benefit.^10^^,^^22^^,^^31^^,^^32^^,^^36^^,^^38^^,^^42^^,^^45^^,^^48^^,^^49^ In the last years, some studies have shown that multimodality treatment can be beneficial for SBAs. Prolonged survival for patients with SBA and IAA has been demonstrated for treatment with capecitabine and oxaliplatin, and fluorouracil, leucovorin and oxaliplatin combination chemotherapy.^15^^,^^21^^,^^63^ In addition, cytoreductive surgery with hyperthermic intraperitoneal chemotherapy was reported to enhance survival in SBA with peritoneal metastases.^64^

The major limitation of this review is the retrospective design of the included studies, with a lack of patient stratification for confounding factors and a lack of specification of treatment modalities in most studies, resulting in clinical heterogeneity of the included studies and risk of bias. Most studies described small patient groups with single-center experience, and provided limited information, in particular on adjusted survival per disease stage and administration of chemo- and/or radiation therapy. Due to variety in the treatment regimens and poor specification of confounding and prognostic factors, bias of these factors could not be investigated in this study.

## Conclusions

Evidence to guide optimal therapeutic outcome is limited due to the rarity of DA. Resection remains the only curative therapeutic option for DA, with equal outcomes after segmental resection and pancreaticoduodenectomy. Based on the included studies, the current use of monotherapy regimens shows no survival benefit, while the optimal approaches for (neo)adjuvant therapy with combined modality therapies are not yet well established. This systematic review and meta-analysis underlines the necessity of improving stratification for high-risk disease, including tumor biology-related factors, subgroup analysis, and targeted approaches, and warrants further research for survival benefit of (neo)adjuvant chemotherapy and metastasectomy in DA.

### Electronic supplementary material

Below is the link to the electronic supplementary material.

Supplementary material 1 (DOCX 42 kb)

## Appendix

See Table 2.

**Table 2:** Overview of the studies describing overall survival specified for resection with curative intent or palliative treatment

Author (year)	Total study population			Resection with curative intent										
	No. of patients	Median survival (months)	5-year survival (%)	No of patients (%)	Age (SD)^a^	Males (%)	Stage 0	Stage I	Stage II	Stage III	Stage IV	Unknown	Median survival (months)	5-year survival (%)
Bakaeen^31^	101	–	37	68 (67)	–	–	2	17	25	22	2	0	–	54
Bhatti^47^	12	18	21	12 (100)	55 (10)	8 (67)		4	3	5		0	18	21
Buchbjerg^2^	71	8	11	28 (39)	–	–		2	11	13	2	0	23	27
Cecchini^32^	169	–	–	103 (61)	–	–						103	44	42
Han^10^	32	–	21	22 (69)	–	–						22	–	32
Hung^33^	23	6	8	11 (48)	64 (13)	7 (64)		4	3	3	1	0	38	17
Hurtuk^44^	52	–	–	35 (67)	–	–						35	34	–
Jiang^34^	187^b^	–	–	128 (68)	–	–						128	57	44
Kaklamanos^12^	63	18	23	37 (59)	–	–		8	11	18		0	31	37
Kawahira^35^	21	–	39	12 (57)	–	–					1	11	59	66
Kelsey^22^	32	–	48	32 (100)	57 (11)	23 (72)		3	18	9	1	1	–	48
Kim^45^	24	–	39	24 (100)	58 (11)	14 (58)		2	10	12		0	–	39
Kim^36^	50	24	32	36 (72)	–	–		7	16	13		0	–	47
Lee^37^	53	–	29	28 (53)	–	–		3	7	18		0	–	44
Lee^38^	70	–	–	41 (59)	–	–		2	21	18		0	25	61
Liang^41^	36	21	–	36 (100)	64 (13)	24 (67)		4	6	26		0	21	–
Malleo^40^	37	70	59	25 (68)	57 (11)	14 (56)		5	6	11	3	0	70	71
Onkendi^11^	124	–	37	99 (80)	–	–						99	38	43
Poultsides^42^	122	–	48	122 (100)	67 (14)	66 (54)		10	28	71	7	6	–	48
Sarela^46^	137	–	–	72 (53)	62 (13)	38 (53)						72	–	71
Solaini^24^	178	44	43	150 (84)	–	–						150	84	51
Struck^48^	30	–	–	30 (100)	61 (10)	22 (73)	2	3	9	16		0	28	53
Swartz^49^	14	41	44	14 (100)	53 (9)	10 (71)				14		0	41	44
Tocchi^39^	47	–	–	25 (53)	–	–						25	38	51
All studies	1685	31^d^	36^d^	1190 (71)			4	74	174	269	17	652	49^d^	48^d^

Author (year)	Palliative treatment								
	No of patients (%)	Age (SD)^a^	Males (%)	Stage II	Stage III	Stage IV	Unknown	Median survival (months)	5-year survival (%)
Bakaeen^31^	33 (33)	–	–				33	–	0
Bhatti^47^									
Buchbjerg^2^	43 (61)	–	–				43	5	0
Cecchini^32^	66 (39)	–	–				66	9	0
Han^10^	10 (31)	–	–				10	–	0
Hung^33^	12^c^ (52)	73 (8)	8 (67)	1	1	10	0	2	0
Hurtuk^44^	17 (33)	–	–				17	–	0
Jiang^34^	59 (32)	–	–				59	7	0
Kaklamanos^12^	26 (41)	–	–		10	5	11	12	0
Kawahira^35^	9 (43)	–	–			9	0	9	0
Kelsey^22^									
Kim^45^									
Kim^36^	14 (28)	–	–		5	8	1	–	0
Lee^37^	25 (47)	–	–				25	8	0
Lee^38^	29 (41)	–	–				29	7	0
Liang^41^									
Malleo^40^	12 (32)	–	7 (58)	1	5	6	0	–	–
Onkendi^11^	25 (20)	–	–				25	6	15
Poultsides^42^									
Sarela^46^	65 (47)	–	37 (57)				65	–	–
Solaini^24^	28 (16)	–	–				28	8	0
Struck^48^									
Swartz^49^									
Tocchi^39^	22 (47)	–	–				22	9	0
All studies	495 (29)			2	21	38	434	8^d^	1^d^

^a^Age: mean in years (range in years)

^b^Long-term analysis

^c^Palliative treatment and supportive therapy

^d^Weighted mean of median survival and 5-year survival of all studies

– Indicates not reported

## References

[1] Legué LM, Bernards N, Gerritse SL (2016). Trends in incidence, treatment and survival of small bowel adenocarcinomas between 1999–2013: a population-based study in The Netherlands. Acta Oncologica..

[2] Buchbjerg T, Fristrup C, Mortensen MB (2015). The incidence and prognosis of true duodenal carcinomas. Surgical Oncology..

[3] Sakae H, Kanzaki H, Nasu J (2017). The characteristics and outcomes of small bowel adenocarcinoma: a multicentre retrospective observational study. Br J Cancer..

[4] Raghav K, Overman MJ (2013). Small bowel adenocarcinomas: existing evidence and evolving paradigms. Nat Rev Clin Oncol..

[5] Haan JC, Buffart TE, Eijk PP (2012). Small bowel adenocarcinoma copy number profiles are more closely related to colorectal than to gastric cancers. Ann Oncol..

[6] Overman MJ, Hu CY, Wolff RA, Chang GJ (2010). Prognostic value of lymph node evaluation in small bowel adenocarcinoma: analysis of the surveillance, epidemiology, and end results database. Cancer..

[7] Tejpar S, Stintzing S, Ciardiello F, et al. Prognostic and predictive relevance of primary tumor location in patients with RAS wild-type metastatic colorectal cancer: retrospective analyses of the CRYSTAL and FIRE-3 trials. JAMA Oncol. Epub 10 Oct 2016. 10.1001/jamaoncol.2016.3797. 10.1001/jamaoncol.2016.3797 PMC7505121 27722750

[8] Jabbour SK, Mulvihill D (2014). Defining the role of adjuvant therapy: ampullary and duodenal adenocarcinoma. Semin Radiat Oncol..

[9] Acharya A, Markar SR, Sodergren MH (2017). Meta-analysis of adjuvant therapy following curative surgery for periampullary adenocarcinoma. Br J Surg..

[10] Han SL, Cheng J, Zhou HZ, Zeng QQ, Lan SH (2008). The surgical treatment and outcome for primary duodenal adenocarcinoma. Journal of Gastrointestinal Cancer..

[11] Onkendi EO, Boostrom SY, Sarr MG (2012). 15-year experience with surgical treatment of duodenal carcinoma: a comparison of periampullary and extra-ampullary duodenal carcinomas. J Gastrointest Surg..

[12] Kaklamanos IG, Bathe OF, Franceschi D, Camarda C, Levi J, Livingstone AS (2000). Extent of resection in the management of duodenal adenocarcinoma. American Journal of Surgery..

[13] Solej M, D’Amico S, Brondino G, Ferronato M, Nano M (2008). Primary duodenal adenocarcinoma. Tumori..

[14] Sakamoto T, Saiura A, Ono Y (2017). Optimal Lymphadenectomy for Duodenal Adenocarcinoma: does the Number Alone Matter?. Ann Surg Oncol..

[15] Kim HS, Shin SJ, Kim JH, Kim H, Choi HJ (2013). Better outcome of xelox chemotherapy in patients with advanced intestinal-type adenocarcinoma of the ampulla of vater. Tohoku Journal of Experimental Medicine..

[16] Overman MJ (2009). Recent advances in the management of adenocarcinoma of the small intestine. Gastrointest Cancer Res..

[17] Wolmark N, Fisher B, Rockette H (1988). Postoperative adjuvant chemotherapy or BCG for colon cancer: results from NSABP protocol C-01. J Natl Cancer Inst..

[18] Wolmark N, Rockette H, Fisher B (1993). The benefit of leucovorin-modulated fluorouracil as postoperative adjuvant therapy for primary colon cancer: results from National Surgical Adjuvant Breast and Bowel Project protocol C-03. J Clin Oncol..

[19] Seymour MT, Maughan TS, Ledermann JA (2007). Different strategies of sequential and combination chemotherapy for patients with poor prognosis advanced colorectal cancer (MRC FOCUS): a randomised controlled trial. Lancet..

[20] Overman MJ, Kopetz S, Wen S (2008). Chemotherapy with 5-fluorouracil and a platinum compound improves outcomes in metastatic small bowel adenocarcinoma. Cancer..

[21] Overman MJ, Varadhachary GR, Kopetz S (2009). Phase II study of capecitabine and oxaliplatin for advanced adenocarcinoma of the small bowel and ampulla of vater. Journal of Clinical Oncology..

[22] Kelsey CR, Nelson JW, Willett CG (2007). Duodenal Adenocarcinoma: patterns of Failure After Resection and the Role of Chemoradiotherapy. International Journal of Radiation Oncology Biology Physics..

[23] Shapiro J, van Lanschot JJ, Hulshof MC (2015). Neoadjuvant chemoradiotherapy plus surgery versus surgery alone for oesophageal or junctional cancer (CROSS): long-term results of a randomised controlled trial. Lancet Oncol..

[24] Solaini L, Jamieson NB, Metcalfe M (2015). Outcome after surgical resection for duodenal adenocarcinoma in the UK. The British journal of surgery..

[25] Moher D, Liberati A, Tetzlaff J, Altman DG (2010). Preferred reporting items for systematic reviews and meta-analyses: the PRISMA statement. Int J Surg..

[26] Cea Compton (2012). AJCC Cancer Staging Atlas: a companion to the Seventh Eiditions of the AJCC Cancer Staging, Manual and Handbook.

[27] DerSimonian R, Laird N (1986). Meta-analysis in clinical trials. Control Clin Trials..

[28] Mantel N, Haenszel W (1959). Statistical aspects of the analysis of data from retrospective studies of disease. J Natl Cancer Inst..

[29] Higgins JP, Thompson SG (2002). Quantifying heterogeneity in a meta-analysis. Stat Med..

[30] Wells GA, Shea B, OConnell D, Peterson J, Welch V, Losos M, et al. The Newcastle–Ottawa Scale (NOS) for assessing the quality of nonrandomised studies in meta-analyses. http://www.ohri.ca/programs/clinical_epidemiology/oxford.asp. Accessed 19 Jul 2017.

[31] Bakaeen FG, Murr MM, Sarr MG, et al. What prognostic factors are important in duodenal adenocarcinoma? Arch Surg. 2000;135(6):635–41; Discussion 641–632. 10.1001/archsurg.135.6.635 10843358

[32] Cecchini S, Correa-Gallego C, Desphande V, et al. Superior prognostic importance of perineural invasion vs. lymph node involvement after curative resection of duodenal adenocarcinoma. J Gastrointest Surg. 2012;16(1):113–20; Discussion 120. 10.1007/s11605-011-1704-6 22005894

[33] Hung FC, Kuo CM, Chuah SK (2007). Clinical analysis of primary duodenal adenocarcinoma: an 11-year experience. Journal of Gastroenterology and Hepatology..

[34] Jiang QL, Huang XH, Chen YT, Zhang JW, Wang CF (2016). Prognostic Factors and Clinical Characteristics of Patients with Primary Duodenal Adenocarcinoma: a Single-Center Experience from China. BioMed Research International..

[35] Kawahira H, Miura F, Saigo K (2011). Survival predictors of patients with primary duodenal adenocarcinoma. Int Surg..

[36] Kim MJ, Choi SB, Han HJ (2014). Clinicopathological analysis and survival outcome of duodenal adenocarcinoma. Kaohsiung Journal of Medical Sciences..

[37] Lee HG, You DD, Paik KY, Heo JS, Choi SH, Choi DW (2008). Prognostic factors for primary duodenal adenocarcinoma. World Journal of Surgery..

[38] Lee SY, Lee JH, Hwang DW, Kim SC, Park KM, Lee YJ (2014). Long-term outcomes in patients with duodenal adenocarcinoma. ANZ journal of surgery..

[39] Tocchi A, Mazzoni G, Puma F (2003). Adenocarcinoma of the third and fourth portions of the duodenum: results of surgical treatment. Archives of Surgery..

[40] Malleo G, Tonsi A, Marchegiani G (2013). Postoperative morbidity is an additional prognostic factor after potentially curative pancreaticoduodenectomy for primary duodenal adenocarcinoma. Langenbecks Arch Surg..

[41] Liang TJ, Wang BW, Liu SI (2012). Number of involved lymph nodes is important in the prediction of prognosis for primary duodenal adenocarcinoma. Journal of the Chinese Medical Association..

[42] Poultsides GA, Huang LC, Cameron JL (2012). Duodenal adenocarcinoma: clinicopathologic analysis and implications for treatment. Ann Surg Oncol..

[43] Cloyd JM, Norton JA, Visser BC, Poultsides GA (2015). Does the extent of resection impact survival for duodenal adenocarcinoma? Analysis of 1,611 cases. Ann Surg Oncol..

[44] Hurtuk MG, Devata S, Brown KM (2007). Should all patients with duodenal adenocarcinoma be considered for aggressive surgical resection?. American Journal of Surgery..

[45] Kim K, Chie EK, Jang JY (2012). Role of adjuvant chemoradiotherapy for duodenal cancer: a single center experience. Am J Clin Oncol..

[46] Sarela AI, Brennan MF, Karpeh MS, Klimstra D, Conlon KC (2004). Adenocarcinoma of the duodenum: importance of accurate lymph node staging and similarity in outcome to gastric cancer. Ann Surg Oncol..

[47] Bhatti ABH, Yosuf MA, Syed AA (2014). Radical surgical management of periampullary duodenal adenocarcinoma: a single institution experience. Journal of the Pakistan Medical Association..

[48] Struck A, Howard T, Chiorean EG, Clarke JM, Riffenburgh R, Cardenes HR (2009). Non-ampullary duodenal adenocarcinoma: factors important for relapse and survival. Journal of Surgical Oncology..

[49] Swartz MJ, Hughes MA, Frassica DA (2007). Adjuvant concurrent chemoradiation for node-positive adenocarcinoma of the duodenum. Arch Surg..

[50] Ecker BL, McMillan MT, Datta J (2017). Adjuvant chemotherapy versus chemoradiotherapy in the management of patients with surgically resected duodenal adenocarcinoma: a propensity score-matched analysis of a nationwide clinical oncology database. Cancer..

[51] Westgaard A, Pomianowska E, Clausen OPF, Gladhaug IP (2013). Intestinal-type and pancreatobiliary-type adenocarcinomas: how does ampullary carcinoma differ from other periampullary malignancies?. Annals of Surgical Oncology..

[52] Chang DK, Jamieson NB, Johns AL (2013). Histomolecular phenotypes and outcome in adenocarcinoma of the ampulla of vater. J Clin Oncol..

[53] Morak MJ, van der Gaast A, Incrocci L (2008). Adjuvant intra-arterial chemotherapy and radiotherapy versus surgery alone in resectable pancreatic and periampullary cancer: a prospective randomized controlled trial. Ann Surg..

[54] Shoji H, Morizane C, Hiraoka N (2014). Twenty-six cases of advanced ampullary adenocarcinoma treated with systemic chemotherapy. Jpn J Clin Oncol..

[55] Yachida S, Wood LD, Suzuki M (2016). Genomic Sequencing Identifies ELF3 as a Driver of Ampullary Carcinoma. Cancer Cell..

[56] Gingras MC, Covington KR, Chang DK (2016). Ampullary Cancers Harbor ELF3 Tumor Suppressor Gene Mutations and Exhibit Frequent WNT Dysregulation. Cell Rep..

[57] de Jong MC, Tsai S, Cameron JL (2010). Safety and efficacy of curative intent surgery for peri-ampullary liver metastasis. J Surg Oncol..

[58] Kannarkatt J, Joseph J, Kurniali PC, Al-Janadi A, Hrinczenko B (2017). Adjuvant Chemotherapy for Stage II Colon Cancer: a Clinical Dilemma. J Oncol Pract..

[59] Andre T, Boni C, Mounedji-Boudiaf L (2004). Oxaliplatin, fluorouracil, and leucovorin as adjuvant treatment for colon cancer. N Engl J Med..

[60] Tol J, Koopman M, Cats A (2009). Chemotherapy, bevacizumab, and cetuximab in metastatic colorectal cancer. N Engl J Med..

[61] Holch JW, Ricard I, Stintzing S, Modest DP, Heinemann V (2017). The relevance of primary tumour location in patients with metastatic colorectal cancer: a meta-analysis of first-line clinical trials. Eur J Cancer..

[62] Petrelli F, Tomasello G, Borgonovo K, et al. Prognostic survival associated with left-sided vs right-sided colon cancer: a systematic review and meta-analysis. JAMA Oncol. Epub 27 Oct 2016. 10.1001/jamaoncol.2016.4227. 10.1001/jamaoncol.2016.4227 27787550

[63] Zaanan A, Costes L, Gauthier M (2010). Chemotherapy of advanced small-bowel adenocarcinoma: a multicenter AGEO study. Ann Oncol..

[64] Legue LM, Simkens GA, Creemers GM, Lemmens V, de Hingh I (2017). Synchronous peritoneal metastases of small bowel adenocarcinoma: insights into an underexposed clinical phenomenon. Eur J Cancer..

