# Supplementary material for: Outcomes and Treatment Options for Duodenal Adenocarcinoma: A Systematic Review and Meta-Analysis
Source: Ann Surg Oncol. 2018 Jun 26;25(9):2681–92. doi: 10.1245/s10434-018-6567-6 (PMC6097725; doi:10.1245/s10434-018-6567-6)
Supplement: Supplementary file 1 — Supplementary material 1 (DOCX 42 kb) [file 10434_2018_6567_MOESM1_ESM.docx]

## SUPPLEMENTARY MATERIAL

*Search strategy*

A systematic literature search was performed in PubMed, EMBASE.com and Wiley/Cochrane Library electronic databases. Databases were searched from inception up to 25 April 2017. The full search strategies for all databases can be found in **Supplementary Table 1.** Duplicates were removed and remaining references were screened on study title and abstract for eligibility based on the predefined inclusion and exclusion criteria (**Supplementary Table 2**). The reference lists of all included papers were screened manually to identify additional relevant papers.

*Eligibility criteria*

All studies reporting on survival for histologically confirmed duodenal adenocarcinoma (DA) or intestinal type ampullary adenocarcinoma (IAA) were eligible for inclusion. Additionally, the reported survival rates had to be specified either per intervention group or per disease stage according to the American Joint Committee on Cancer (AJCC) TNM system.^25^ Studies involving a subset of relevant patients were included if data were specified per subgroup and contained more than ten relevant patients. Conference abstracts, case reports (i.e. sample size fewer than ten patients), no specified survival data, review articles, and studies published in languages other than English were excluded.

*Data extraction and analysis*
Data extraction items included study year, study period, study design, study population, patient characteristics, interventions, disease stage, survival outcomes per disease stage and intervention group, follow-up period and author’s key conclusions. For age, mean values and standard deviation (SD) were displayed as reported originally or calculated from the published raw data if possible.^26^ For survival data, median survival and 5-year survival were displayed as reported originally or survival rates were calculated based on raw data.^27,28^ Outcomes were summed and weighted averages of the means were determined.

*Statistical analysis*
Statistical analyses included pooling of studies and was confined to studies reporting 5-year OS in order to maximize the number of eligible studies. Adjusted estimates of survival were not reported sufficiently to be included for meta-analysis. The number of events after 5 years were compared between groups of interest and the odds ratios (ORs) for death with their associated 95% confidence interval (C.I.) were calculated from the raw extracted data, including the number of events in each group and the number of included patient in each group. The Mantel-Haenszel method was used to calculate the weighted pooled OR and their associated 95% C.I. for dichotomous data under the random effects model.^29,30^ Forest plots were used for graphic display of the results. P-values below 0.05 for the test of survival difference between the groups were considered statistically significant. It should be noted that pooled analysis were assessed as binary variables (curative treatment versus palliative treatment, adjuvant versus no adjuvant therapy, and involvement of lymph nodes versus no lymph node involvement). Subgroup analyses (i.e. per tumor stage and specific type of treatment) could not be taken into consideration due to the lack of sufficient specified data. Statistical heterogeneity was estimated using the Cochrane’s Q and *I^2^* statistics.^31^ *I^2^* values above 50 were considered as substantial heterogeneity. Statistical analyses were performed with Review Manager (RevMan) version 5.3 (The Cochrane Collaboration, Copenhagen, Denmark). Outcomes were reviewed qualitatively if a meta-analysis could not be performed.

*Assessment of Methodological Quality*
The Newcastle – Ottawa quality assessment scale (0 – 9 points) was implemented to assess the quality and risk of bias of the included studies.^32^ A follow-up duration of at least 3 years was considered sufficient, and the maximum loss to follow-up of less than 10% was awarded a point. Studies with scores below 4 were considered to have a high risk of bias, those with scores of 4 - 6 to have an intermediate risk of bias, and those with scores of 7 or more to have a low risk of bias.

Supplementary Table 1 **–** Full search strategy for all three databases.

**PubMed Session Results (25 Apr 2017)**

| Search | Query | Items found |
| --- | --- | --- |
| [#10](https://www.ncbi.nlm.nih.gov/pubmed) | #7 AND #8 AND #9 | [2,948](https://www.ncbi.nlm.nih.gov/pubmed/?cmd=HistorySearch&querykey=10) |
| [#9](https://www.ncbi.nlm.nih.gov/pubmed) | "Therapeutics"[Mesh] OR therapeutic*[tiab] OR therapy[tiab] OR therapies[tiab] OR treatment[tiab] OR treatments[tiab] OR "Surgical Procedures, Operative"[Mesh] OR operative surgical procedure*[tiab] OR operative procedure*[tiab] OR pancreaticoduodenectom*[tiab] OR pancreatoduodenectom*[tiab] OR duodenopancreatectom*[tiab] OR PPPD[tiab] OR whipple[tiab] OR adjuvant chemotherap*[tiab] OR radiochemotherap*[tiab] OR chemoradiotherap*[tiab] OR radio-chemotherap*[tiab] OR chemo-radiotherap*[tiab] OR chemotherap*[tiab] OR radiotherap*[tiab] OR metastasectom*[tiab] OR papillectom*[tiab] OR ampullectom*[tiab] OR resectabilit*[tiab] OR resectable[tiab] OR resection*[tiab] OR ablation technique*[tiab] OR catheter ablat*[tiab] OR RFA[tiab] OR radiofrequency ablat*[tiab] OR microwave ablat*[tiab] OR ultrasound ablat*[tiab] | [8,817,897](https://www.ncbi.nlm.nih.gov/pubmed/?cmd=HistorySearch&querykey=9) |
| [#8](https://www.ncbi.nlm.nih.gov/pubmed) | (late[tiab] AND (effect[tiab] OR effects[tiab] OR complication*[tiab] OR onset[tiab])) OR sequela*[tiab] OR "long term"[tiab] OR longterm[tiab] OR following[tiab] OR "follow up"[tiab] OR followup[tiab] OR surviv*[tiab] OR "Survivors"[Mesh] OR adulthood[tiab] OR lifelong[tiab] OR "life long"[tiab] OR permanent[tiab] OR cured[tiab] OR (extended[tiab] AND period*[tiab]) OR mortality[tiab] OR "Longitudinal Studies"[Mesh] OR "Time factors"[MeSH] OR (adult[tiab] AND "treatment outcome"[tiab]) OR Psychology[sh] OR Complications[sh] OR "Risk factors"[MeSH] OR "Survival Analysis"[Mesh] OR Kaplan Meier[tiab] OR product-limit method*[tiab] | [6,975,003](https://www.ncbi.nlm.nih.gov/pubmed/?cmd=HistorySearch&querykey=8) |
| [#7](https://www.ncbi.nlm.nih.gov/pubmed) | #3 OR #6 | [7,141](https://www.ncbi.nlm.nih.gov/pubmed/?cmd=HistorySearch&querykey=7) |
| [#6](https://www.ncbi.nlm.nih.gov/pubmed) | (#1 AND #4) OR #5 | [2,894](https://www.ncbi.nlm.nih.gov/pubmed/?cmd=HistorySearch&querykey=6) |
| [#5](https://www.ncbi.nlm.nih.gov/pubmed) | "Ampulla of Vater/pathology"[Mesh] | [1,438](https://www.ncbi.nlm.nih.gov/pubmed/?cmd=HistorySearch&querykey=5) |
| [#4](https://www.ncbi.nlm.nih.gov/pubmed) | periampulla*[tiab] OR ampullar*[tiab] OR peri ampulla[tiab] OR hepatopancreatic ampulla[tiab] OR Ampulla of Vater[tiab] OR Vater Ampulla[tiab] OR Vater's Ampulla[tiab] OR duodenal papilla[tiab] OR duodenal ampulla[tiab] | [8,443](https://www.ncbi.nlm.nih.gov/pubmed/?cmd=HistorySearch&querykey=4) |
| [#3](https://www.ncbi.nlm.nih.gov/pubmed) | #1 AND #2 | [5,749](https://www.ncbi.nlm.nih.gov/pubmed/?cmd=HistorySearch&querykey=3) |
| [#2](https://www.ncbi.nlm.nih.gov/pubmed) | "Duodenal Neoplasms"[Mesh] OR "Duodenum"[Mesh] OR duodenum[tiab] OR duodenal[tiab] | [98,088](https://www.ncbi.nlm.nih.gov/pubmed/?cmd=HistorySearch&querykey=2) |
| [#1](https://www.ncbi.nlm.nih.gov/pubmed) | "Adenocarcinoma"[Mesh] OR malignant adenoma*[tiab] OR adenocarcinoma*[tiab] OR adeno carcinoma*[tiab] OR glandular carcinoma*[tiab] | [370,431](https://www.ncbi.nlm.nih.gov/pubmed/?cmd=HistorySearch&querykey=1) |

**Embase.com Session Results (25 Apr 2017)**

| Search | Query | Items found |
| --- | --- | --- |
| [#10](https://www.ncbi.nlm.nih.gov/pubmed) | #7 AND #8 AND #9 | 3,207 |
| [#9](https://www.ncbi.nlm.nih.gov/pubmed) | 'therapy'/exp OR therapeutic*:ab,ti OR therapy:ab,ti OR therapies:ab,ti OR treatment:ab,ti OR treatments:ab,ti OR 'surgery'/exp OR 'operative surgical procedure*':ab,ti OR 'operative procedure*':ab,ti OR pancreaticoduodenectom*:ab,ti OR pancreatoduodenectom*:ab,ti OR duodenopancreatectom*:ab,ti OR PPPD:ab,ti OR whipple:ab,ti OR 'adjuvant chemotherap*':ab,ti OR radiochemotherap*:ab,ti OR chemoradiotherap*:ab,ti OR 'radio-chemotherap*':ab,ti OR 'chemo-radiotherap*':ab,ti OR chemotherap*:ab,ti OR radiotherap*:ab,ti OR metastasectom*:ab,ti OR papillectom*:ab,ti OR ampullectom*:ab,ti OR resectabilit*:ab,ti OR resectable:ab,ti OR resection*:ab,ti OR 'ablation technique*':ab,ti OR 'catheter ablat*':ab,ti OR RFA:ab,ti OR 'radiofrequency ablat*':ab,ti OR 'microwave ablat*':ab,ti OR 'ultrasound ablat*':ab,ti | 12,359,546 |
| [#8](https://www.ncbi.nlm.nih.gov/pubmed) | (late:ab,ti AND (effect:ab,ti OR effects:ab,ti OR complication*:ab,ti OR onset:ab,ti)) OR sequela*:ab,ti OR 'long term':ab,ti OR longterm:ab,ti OR following:ab,ti OR 'follow up':ab,ti OR followup:ab,ti OR surviv*:ab,ti OR 'survivor'/exp OR adulthood:ab,ti OR lifelong:ab,ti OR 'life long':ab,ti OR permanent:ab,ti OR cured:ab,ti OR (extended:ab,ti AND period*:ab,ti) OR mortality:ab,ti OR 'longitudinal study'/exp OR 'time factor'/exp OR (adult:ab,ti AND 'treatment outcome':ab,ti) OR 'risk factor'/exp OR 'survival analysis'/exp OR 'Kaplan Meier':ab,ti OR 'product-limit method*':ab,ti | 5,594,974 |
| [#7](https://www.ncbi.nlm.nih.gov/pubmed) | #3 OR #6 | 11,425 |
| [#6](https://www.ncbi.nlm.nih.gov/pubmed) | (#1 AND #4) OR #5 | 7,901 |
| [#5](https://www.ncbi.nlm.nih.gov/pubmed) | 'ampulla of Vater'/exp | 6,343 |
| [#4](https://www.ncbi.nlm.nih.gov/pubmed) | periampulla*:ab,ti OR ampullar*:ab,ti OR 'peri ampulla':ab,ti OR 'hepatopancreatic ampulla':ab,ti OR (Ampulla NEAR/3 Vater*):ab,ti OR 'duodenal papilla':ab,ti OR 'duodenal ampulla':ab,ti | 11,249 |
| [#3](https://www.ncbi.nlm.nih.gov/pubmed) | #1 AND #2 | 4,700 |
| [#2](https://www.ncbi.nlm.nih.gov/pubmed) | 'duodenum tumor'/exp OR 'duodenum'/exp OR duodenum:ab,ti OR duodenal:ab,ti | 111,255 |
| [#1](https://www.ncbi.nlm.nih.gov/pubmed) | 'adenocarcinoma'/exp OR 'malignant adenoma*':ab,ti OR adenocarcinoma*:ab,ti OR 'adeno carcinoma*':ab,ti OR 'glandular carcinoma*':ab,ti | 234,519 |

**Wiley / Cochrane Library Session Results (25 Apr 2017)**

| Search | Query | Items found |
| --- | --- | --- |
| [#10](https://www.ncbi.nlm.nih.gov/pubmed) | #7 AND #8 AND #9 | 115 |
| [#9](https://www.ncbi.nlm.nih.gov/pubmed) | therapeutic*:ab,ti,kw or therapy:ab,ti,kw or therapies:ab,ti,kw or treatment:ab,ti,kw or treatments:ab,ti,kw or "operative surgical procedure*":ab,ti,kw or "operative procedure*":ab,ti,kw or pancreaticoduodenectom*:ab,ti,kw or pancreatoduodenectom*:ab,ti,kw or duodenopancreatectom*:ab,ti,kw or PPPD:ab,ti,kw or whipple:ab,ti,kw or "adjuvant chemotherap*":ab,ti,kw or radiochemotherap*:ab,ti,kw or chemoradiotherap*:ab,ti,kw or "radio-chemotherap*":ab,ti,kw or "chemo-radiotherap*":ab,ti,kw or chemotherap*:ab,ti,kw or radiotherap*:ab,ti,kw or metastasectom*:ab,ti,kw or papillectom*:ab,ti,kw or ampullectom*:ab,ti,kw or resectabilit*:ab,ti,kw or resectable:ab,ti,kw or resection*:ab,ti,kw or "ablation technique*":ab,ti,kw or "catheter ablat*":ab,ti,kw or RFA:ab,ti,kw or "radiofrequency ablat*":ab,ti,kw or "microwave ablat*":ab,ti,kw or "ultrasound ablat*":ab,ti,kw | 605,179 |
| [#8](https://www.ncbi.nlm.nih.gov/pubmed) | (late:ab,ti,kw and (effect:ab,ti,kw or effects:ab,ti,kw or complication*:ab,ti,kw or onset:ab,ti,kw)) or sequela*:ab,ti,kw or "long term":ab,ti,kw or longterm:ab,ti,kw or following:ab,ti,kw or "follow up":ab,ti,kw or followup:ab,ti,kw or surviv*:ab,ti,kw or adulthood:ab,ti,kw or lifelong:ab,ti,kw or "life long":ab,ti,kw or permanent:ab,ti,kw or cured:ab,ti,kw or (extended:ab,ti,kw and period*:ab,ti,kw) or mortality:ab,ti,kw or "longitudinal stud*":ab,ti,kw or "time factor*":ab,ti,kw or (adult:ab,ti,kw and "treatment outcome":ab,ti,kw) or "risk factor" *:ab,ti,kw or "Kaplan Meier":ab,ti,kw or "product-limit method*":ab,ti,kw | 408,445 |
| [#7](https://www.ncbi.nlm.nih.gov/pubmed) | #3 OR #6 | 186 |
| [#6](https://www.ncbi.nlm.nih.gov/pubmed) | (#1 AND #4) OR #5 | 156 |
| [#5](https://www.ncbi.nlm.nih.gov/pubmed) | (Ampulla near/3 Vater*):ab,ti,kw | 128 |
| [#4](https://www.ncbi.nlm.nih.gov/pubmed) | periampulla*:ab,ti,kw or ampullar*:ab,ti,kw or "peri ampulla":ab,ti,kw or "hepatopancreatic ampulla":ab,ti,kw or "duodenal papilla":ab,ti,kw or "duodenal ampulla":ab,ti,kw | 229 |
| [#3](https://www.ncbi.nlm.nih.gov/pubmed) | #1 AND #2 | 41 |
| [#2](https://www.ncbi.nlm.nih.gov/pubmed) | duodenum:ab,ti,kw or duodenal:ab,ti,kw | 5,857 |
| [#1](https://www.ncbi.nlm.nih.gov/pubmed) | "malignant adenoma*":ab,ti,kw or adenocarcinoma*:ab,ti,kw or "adeno carcinoma*":ab,ti,kw or "glandular carcinoma*":ab,ti,kw | 5,330 |

**Supplementary Table 2 -** Inclusion and exclusion criteria of the selected studies

| Inclusion criteria | | **Exclusion criteria** | | |  |  |  |
| --- | --- | --- | --- | --- | --- | --- | --- |
| Study type | | | | |  |  |  |
| - Studies written in English  - Human studies | - Animal studies  - Meta-analysis | | | |  |  |  |
| - Randomized controlled trial | - | | Systematic review | | |  |  |
| - Non-randomized controlled trial | - | | Case series with less than 10 patients | | | - | |
| - Prospective cohort study | - | | Conference abstract | | |  | |
| - Retrospective cohort study |  | |  | | |  |  |
| - Case-control study |  | |  | | |  |  |
| - Case series with 10 or more patients  - Studies involving only a subset of relevant patients will be included if data is subdivided and contains ≥10 relevant patients |  | |  | | |  |  |
| Type of participants | | | | |  |  |  |
| - Histopathological diagnosis of duodenal adenocarcinoma or intestinal type of Vater adenocarcinoma (including signet cell carcinoma and mucinous adenocarcinoma) - Primary tumor  - Disease stages, or T and N classification, or treatment modality specified for the included patients  - Age ≥ 18 year  - Male and female | **-**  **-** | | | Benign disease  - Secondary tumor / metastasis of other primary tumor | | |  |
| Type of intervention | | | | |  |  |  |
| - Surgical intervention, including resection with curative intent and palliative surgery  - Neoadjuvant therapy - Adjuvant therapy, including chemotherapy, radiotherapy and chemoradiation  - Palliative treatment, including chemotherapy, radiotherapy, and chemoradiation  - Local therapy of metastasis |  | | |  | | |  |
| Primary outcome | | | | | | |  |
| - Overall survival; specified:   1. Per disease stage 2. Per treatment modality |  | | |  | | |  |
